# Serum Response Factor Controls CYLD Expression via MAPK Signaling Pathway

**DOI:** 10.1371/journal.pone.0019613

**Published:** 2011-05-05

**Authors:** Gang Liang, Kristofer Ahlqvist, Rajeswararao Pannem, Guido Posern, Ramin Massoumi

**Affiliations:** 1 Department of Laboratory Medicine, Lund University, UMAS, Malmö, Sweden; 2 AG Regulation of Gene Expression, Department of Molecular Biology, Max Planck Institute of Biochemistry, Martinsried, Germany; Ohio State University, United States of America

## Abstract

Tumor suppressor gene CYLD is a deubiquitinating enzyme which negatively regulates various signaling pathways by removing the lysine 63-linked polyubiquitin chains from several specific substrates. Loss of CYLD in different types of tumors leads to either cell survival or proliferation. In this study we demonstrate that lack of CYLD expression in CYLD−/− MEFs increases proliferation rate of these cells compared to CYLD+/+ in a serum concentration dependent manner without affecting cell survival. The reduced proliferation rate in CYLD+/+ in the presence of serum was due to the binding of serum response factor (SRF) to the serum response element identified in the CYLD promoter for the up-regulation of CYLD levels. The serum regulated recruitment of SRF to the CYLD promoter was dependent on p38 mitogen-activated protein kinase (MAPK) activity. Elimination of SRF by siRNA or inhibition of p38 MAPK reduced the expression level of CYLD and increased cell proliferation. These results show that SRF acts as a positive regulator of CYLD expression, which in turn reduces the mitogenic activation of serum for aberrant proliferation of MEF cells.

## Introduction

CYLD is a deubiquitinating enzyme (DUB) which is absent or strongly down regulated in different types of human cancer. Cylindromatosis was the first described skin cancer caused by mutation in the CYLD gene and subsequent loss of heterozygosity [1]. Beside cylindromatosis, the expression of CYLD is dramatically down-regulated in other types of human cancer such as melanoma [2], cervix cancer [3], colon cancer [4], [5] and multiple myeloma [6], [7]. Removal of the lysine 63 (K63) linked polyubiquitin chains from different CYLD substrates including Bcl-3, TRAF2 and TAK1 interferes and modulates different signaling pathways such as NF-κB, JNK and p38 MAPK [8]–[9]. In vivo, CYLD deficient mice were highly sensitive to chemically induced skin tumors and developed significantly larger and faster-growing skin papillomas [10]. This effect was attributable to the elevated expression of cyclin D1 which caused an increase in the proliferation rate of CYLD-deficient keratinocytes than wild-type controls [10]. Even though previous studies has been highlighting the importance of post translational modifications of CYLD such as phosphorylation [11], [12] and ubiquitination [13] for its tumor suppressor function, at the present there is a lack of knowledge of how CYLD transcription is regulated and by which signaling pathway this regulation occurs in non-transformed primary cells.

Serum response factor (SRF) is a member of the highly conserved MADS box family of transcription factors which regulates the expression of immediate early genes. SRF binds to its specific promoter sequence (CArG box), which is often located within a slightly larger serum response element (SRE) [14], [15], [16]. SRF is conserved from flies to humans and is encoded by a single gene that is widely expressed. Functional SRF binding sites have been identified in the promoters of many genes which encode signaling molecules, transcription factors and many cytoskeletal components, such as c-fos and actin. One class of SRF co-activators are activated by the MAPK pathway in response to mitogenic and stress stimuli [17]. A second class of SRF specific co-activators the MAL/MRTF proteins are released from a repressive complex with G-actin upon induced changes in actin dynamics [18]. Activated MRTF family members inhibit cell proliferation, probably through transcriptional up-regulation of several anti-proliferative targets [19], [20], [21]. Considerable alterations in the DNA binding activity of SRF upon serum induction are either not observed or are associated with additional events such as phosphorylation, depending on the cell type, the stimulus and the target gene [22], [23], [24], [25].

In the course of characterizing of the properties of mouse embryonic fibroblasts (MEFs) derived from wild-type and CYLD−/− mice, we made the surprising observation that the CYLD−/− MEFs had elevated proliferation rate compared to the CYLD+/+ in the presence of serum. We found a novel role for how CYLD expression is regulated by serum through SRF activation which caused a reduction in cell proliferation. Serum promoted recruitment of SRF to the SRE sites located in the CYLD promoter through p38 MAPK activation. These results implicate reduced proliferation rate of CYLD expressing MEF cells mediated by p38 and SRF activation, important for balancing proliferation and homoestasis of MEF cells.

## Results

### CYLD-deficient murine embryonic fibroblasts grow much faster than wild-type cells

**Figure 1:**
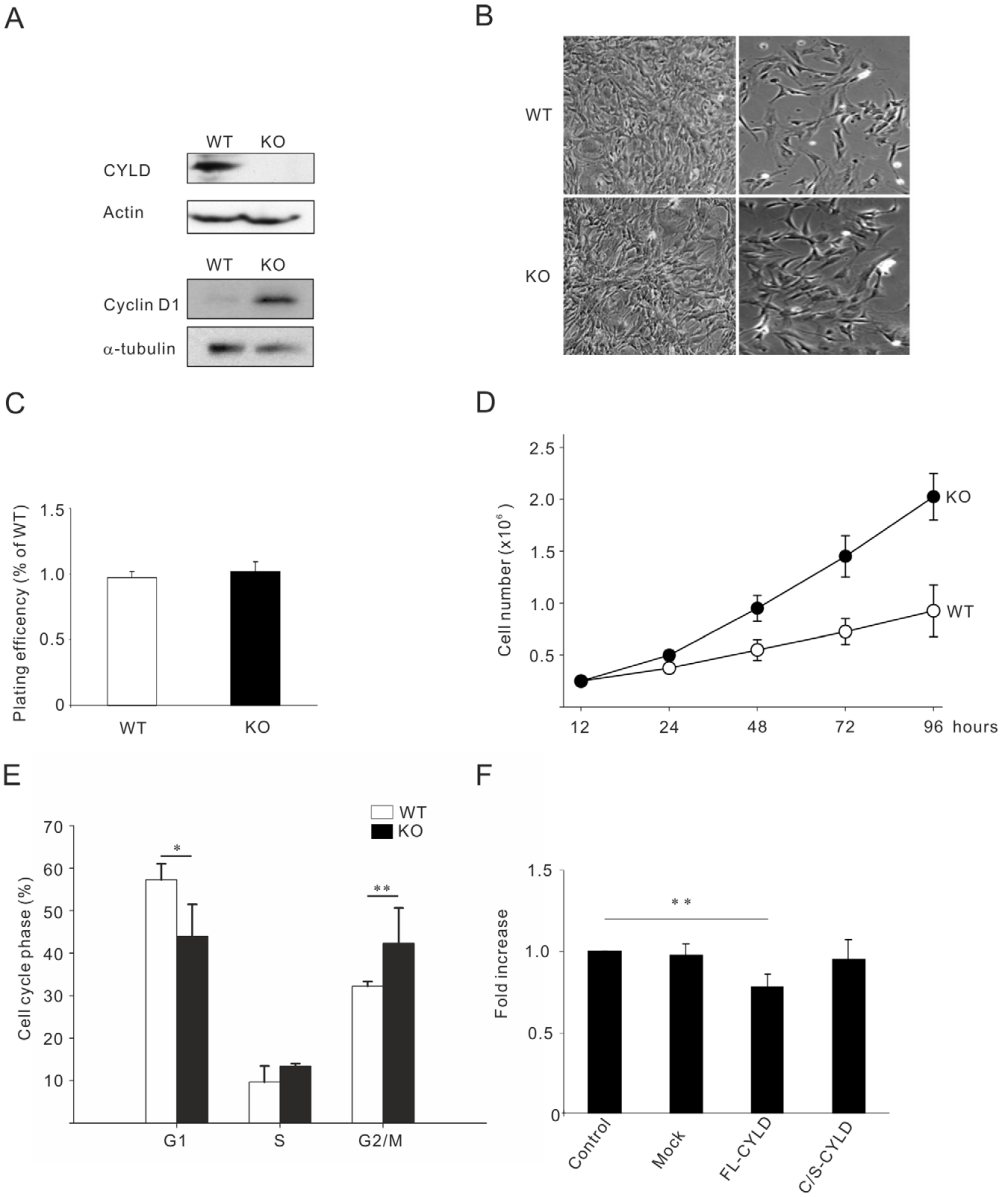
Increased proliferation rate of primary CYLD−/− compared to CYLD +/+ MEFs. (**A**). Mouse embryonic fibroblast (MEF) protein extracts from primary wild-type (WT) and CYLD knockout (KO) probed with antibodies against CYLD or actin. (**B**). Morphology (left panels) and spreading (right panels) of CYLD-WT and KO MEF cells. (**C**). Plating efficiency of CYLD-WT and KO MEF cells measured as total number of adherent cells (12 hours) expressed as the percentage of total cells plated. (**D**). Measurement of the growth rate of MEFs cultured in the presence of 10% fetal calf serum (FCS) by cell counting over a period of 96 hours. (**E**). Cell cycle analysis of WT and KO MEFs after serum depravation (24 hours) followed by re-addition of 10% fetal calf serum for 24 hours. (**F**). Cell counting of CYLD−/− MEFs transiently transfected with mock, full length (FL-CYLD) or catalytic inactive mutant of CYLD (C/S-CYLD) expression plasmid after 48 hours in the presence of 10% serum.

Primary CYLD+/+ and CYLD−/− MEFs, obtained from littermate embryos were isolated (Figure 1A, upper panels) and compared for their in vitro growth properties. To reduce the individual variability, MEFs isolated from embryos of the same litter and having the same genotype were pooled together and independent pools were analyzed in different experiments. The early-passage (P2) MEFs which were grown in the presence of 10% fetal calf serum (FCS), were found to exhibit the same morphology (Figure 1B, left panels), spreading (Figure 1B, right panels) and plating efficiency (Figure 1C). Surprisingly, measurement of the growth rate of MEFs grown in the presence of 10% fetal calf serum over a period of 96 hours showed that CYLD deficient cells grew significantly faster than wild-type cells using cell counting assay (Figure 1D) and expressed high levels of cyclin D1 (Figure 1A lower panels). The same result was obtained when three other proliferation assays (BrdU, MTS and Almar blue) were employed or when we used later passage of MEFs (Passage 5) (Data not shown). In accordance with the difference in proliferation rate, we investigated the duration of the cell cycle by using serum starvation to synchronize the cells and then analysed cell-cycle progression after 24 hours. We found an increase in the amount of CYLD+/+ cells in G1 phase and a decrease in G2/M phase compared to the CYLD−/− MEFs (Figure 1E). To determine whether the increase in the proliferation rate observed in CYLD deficient cells depends on the deubiquitinating activity of CYLD, we transiently transfected MEF cells with full-length and catalytically inactive mutant of CYLD (CYLD^C/S^). Full length CYLD but not CYLD^C/S^ could significantly reduce proliferation rate of CYLD−/− MEFs after 72 hours (Figure 1F) indicating that the deubiquitinating activity of CYLD is required for the observed phenotype.

### The increases proliferation rate of CYLD−/− MEFs is dependent on serum concentration

**Figure 2:**
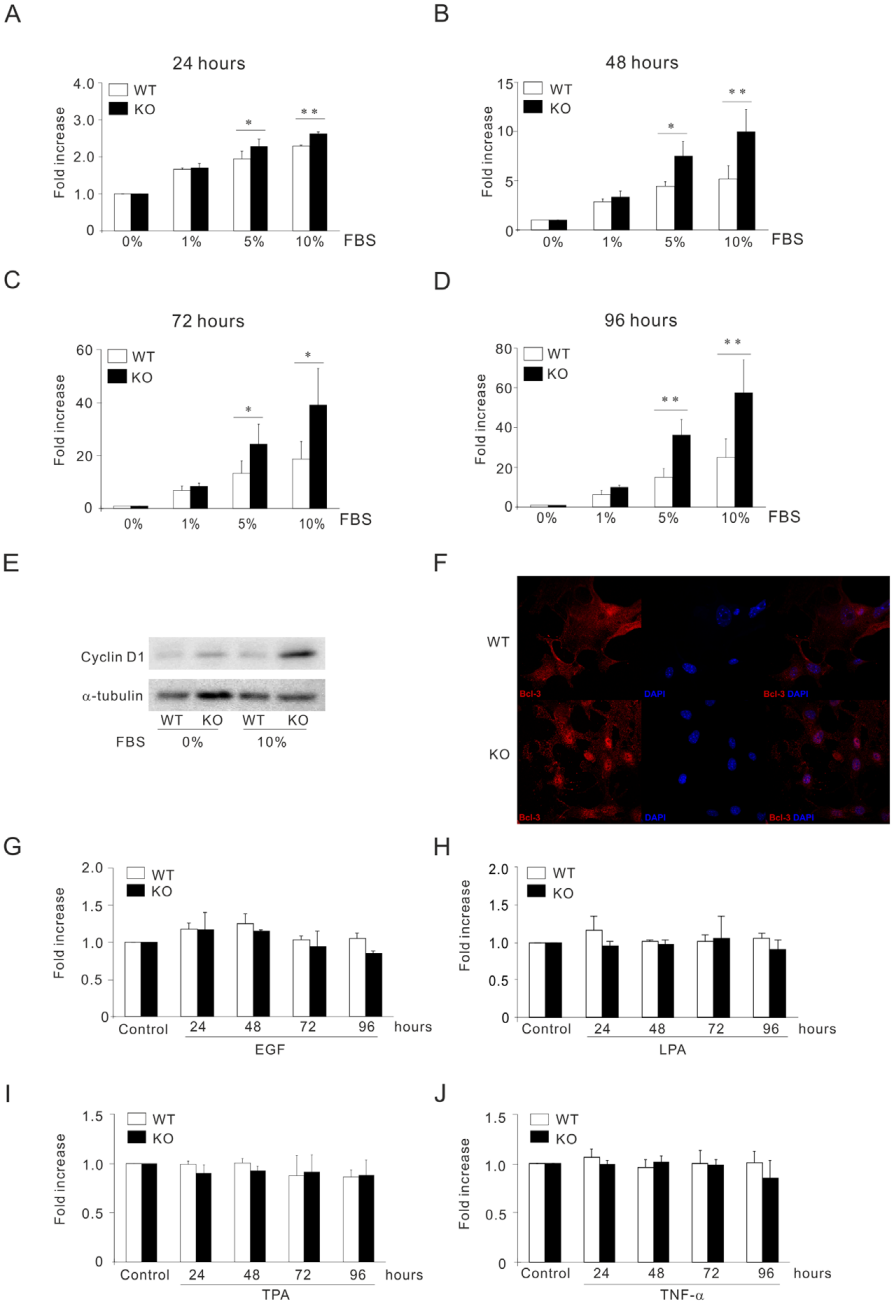
Serum concentration dependent proliferation of CYLD+/+ and CYLD−/− primary MEFs. (**A–D**). Measurement of the growth rate of MEFs by cell counting after serum withdrawal (24 hours) followed by re-addition of FCS: 0, 1, 5 and 10% over a period of 24–96 hours. (**E**). Analysis of the levels of cyclin D1 and tubulin in primary CYLD+/+ compared to CYLD −/− MEFs in the absence (0%) or presence of 10% FCS for 48 hours. (**F**). Confocal plane of Bcl-3 (red) and DAPI (blue) in CYLD+/+ (upper) and CYLD−/− (lower) MEFs after serum withdrawal (24 hours) followed by re-addition of 10% FCS for 24 hours. (**G–J**). Measurement of the growth rate of MEFs by cell counting treated with serum free for 24 hours before re-addition of 1% FCS together with EGF (100 ng/ml), LPA (1 µM), TPA (100 nM) and or TNF-α (100 ng/ml) over a period of 24–96 hours.

As the doubling time for CYLD deficient MEF cells were shorter compared to wild-type MEF in the presence of 10% serum, we hypothesized that this effect might be dependent on the concentration of serum. To investigate this, wild-type and knockout MEFs were cultured in media with different concentrations of serum (0, 1, 5 and 10%) and at different time points (24, 48, 72 and 96 h). Interestingly, we could observe no differences in the proliferation rates (24–96 h) between the wild-type and knockout cells in the absence of serum, nor in the presence of 1% (Figure 2A–D). Whereas, in the presence of 5% or 10% serum significant differences in proliferation rate between wild-type and knockout cells could be observed (Figure 2A–D). Together these results suggest that serum contribute to the differences in the proliferation rate between wild-type and knockout cells. The best characterized of the cyclins that is inducible after serum induction as initiator for the G1 phase of the cell cycle is cyclin D1. We detected low levels of cyclin D1 in both wild-type and knockout cells in the absence of serum (Figure 2E), whereas an accumulation of cyclin D1 was observed in knockout compared to the wild-type cells cultured in 10% serum (Figure 2E) supporting the phenotype observed for the rapid growth rate in CYLD−/− cells compared to the wild-type cells. We have reported earlier that CYLD by retaining Bcl-3 in the cytoplasm reduces the expression of cyclin-D1 and consequently decreases the proliferation of keratinocytes [10] and melanoma cells [26]. Interestingly, in the presence of serum, we found an increase in the number of nuclear localized Bcl-3 in CYLD−/− cells compared to CYLD+/+ MEF cells using confocal microscopy (Figure 2F).

**Figure 3:**
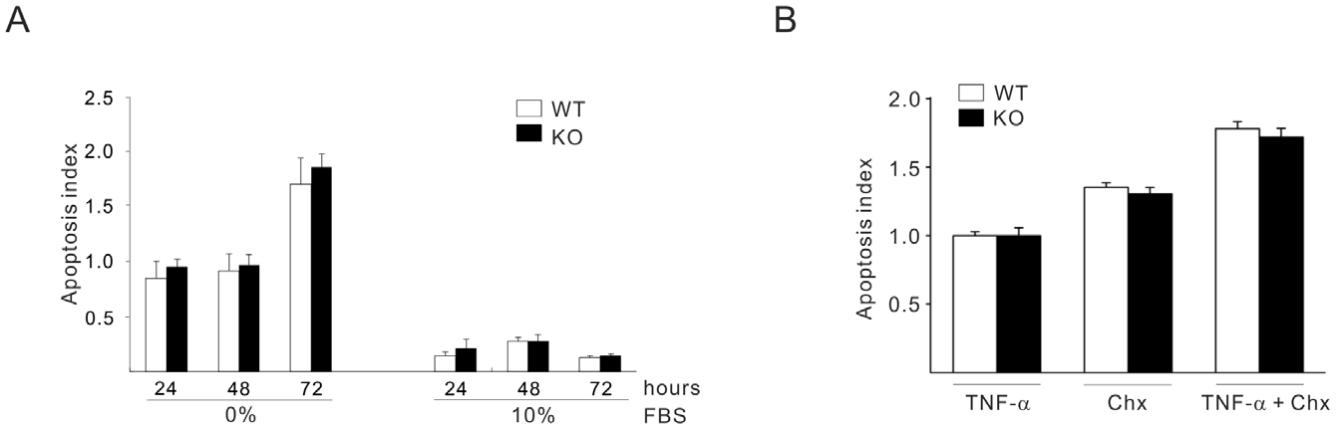
Effects of TNF-α mediated apoptosis in CYLD +/+ and CYLD−/− MEFs. (**A**). The apoptotic cells were analyzed by detecting the gradual degradation of internucleosomal DNA with the DNA binding fluorescent dye propidium iodide in WT and CYLD-KO MEF cells in the absence or presence of 10% FCS over a period of 24–96 hours. (**B**). The apoptotic cells were analyzed by detecting the gradual degradation of internucleosomal DNA with the DNA binding fluorescent dye propidium iodide in WT and CYLD-KO MEF cells treated with TNF-α (100 ng/ml) for 12 hours, cyclohexamide (10 µg/ml) for 12 hours or a combination of TNF-α and cyclohexamide (100 ng/ml respective 10 µg/ml) for 12 hours.

Since epidermal growth factor (EGF) and lysophosphatidic acid (LPA) in serum are strong mitotic stimuli, we investigated proliferation properties of MEFs in the presence of these agonists in 1% concentration of serum. Treatment with 10 ng/ml EGF (Figure 2G) or 1 µM of LPA (Figure 2H) were unable to increase proliferation rate of CYLD−/− MEFs compared to CYLD+/+. Treatment of MEFs with either 12-O-tetradecanoylphorbol-13 acetate (TPA) (Figure 2I) or TNF-α (Figure 2J) which was shown earlier to regulate CYLD mediated signal transduction [10], [27], were unable to increase the proliferation rate of CYLD−/− MEFs compared to CYLD+/+ over a period of 24–96 hours (Figure 2I, 2J). To ensure that the increase proliferation observed in CYLD−/− MEFs was not due to the increase cell death of CYLD+/+ MEFs, we performed apoptosis assay in the absence or presence of serum. As anticipated, in the absence of serum more apoptotic cells could be observed compared to cells treated with 10% of serum (Figure 3A). Interestingly, absent or presence of serum did not induce significant differences in the number of apoptotic cells in MEF-CYLD+/+ compared to CYLD−/− over a period of 24–72 hours (Figure 3A). Since it was shown earlier that CYLD knockdown is sensitive to TNF-α mediated apoptosis in different transformed cell lines [27], [28], here we treated CYLD+/+ and CYLD −/− cells with either TNF-α, cyclohexamide or a combination of these two. Figure 3B, shows that treatment with TNF-α, alone or in combination with cyclohexamide is unable to affect the number of viable cells between wild-type and knockout cells. These results suggest that loss of CYLD mediates proliferation and not survival in MEF cells.

### CYLD gene expression is regulated rapidly at the transcriptional level by serum

**Figure 4:**
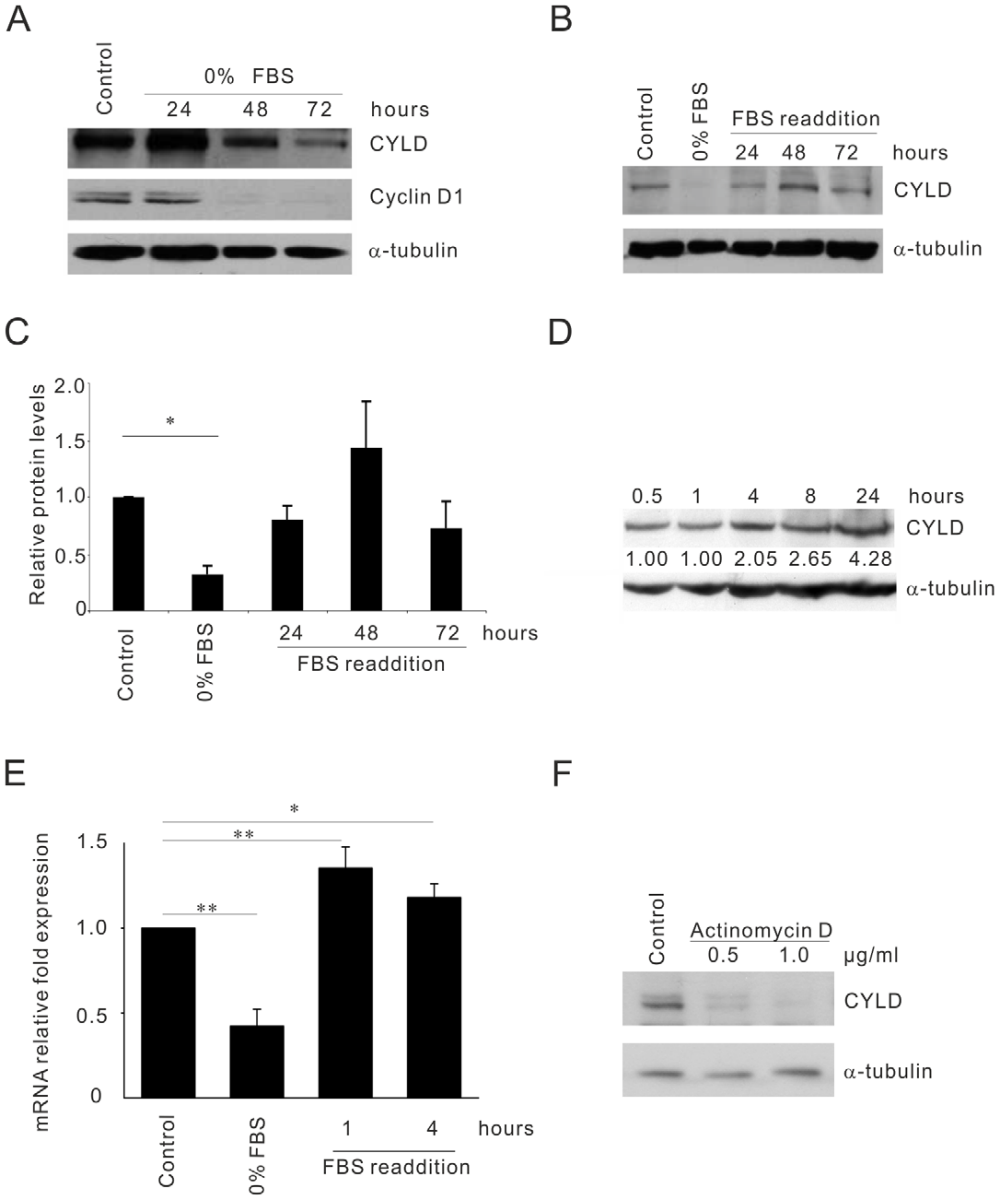
CYLD gene expression is regulated at the transcriptional level by serum. (**A**). Analysis of the levels of CYLD, cyclin D1 and tubulin in primary CYLD+/+ MEFs in 10% FCS (control) or serum deprived cells over a period of 24–72 hours. (**B–C**). Analysis of the levels of CYLD and tubulin in primary CYLD+/+ MEFs in 10% FCS (control) or serum deprived cells for 24 hours (0%) or re-addition of FCS (10%) to the cells over a period of 24–72 hours. (**D**). Analysis of the levels of CYLD and tubulin in primary CYLD+/+ MEFs in 10% FCS over a period of 0.5–24 hours. (**E**). CYLD gene expression by using qRT-PCR upon withdrawal (0% FCS) or re-addition of serum to the cell cultures for 1 or 4 hours. (**F**). Analysis of the levels of CYLD and tubulin in primary CYLD+/+ MEFs after serum deprivation 24 hours before re-addition of 10% FCS (control) or 10% FCS together with 0.5–1.0 µg/ml actinomycin D for 12 hours.

To investigate whether CYLD protein level is dependent on the concentration of serum in culture media, CYLD+/+ MEF cells were cultured under serum deprived conditions. This treatment resulted in a drop in the CYLD protein level (Figure 4A) which was recovered upon serum re-addition and peaked at approximately after 48 hours (Figure 4B, 4C). To investigate how rapidly inducible CYLD expression occur post serum induction, cells were serum starved for 24 hours before serum re-addition. As early as four hours we could detect a two-fold increase in CYLD protein levels (Figure 4D). This regulation appeared to take place at the transcriptional level, since the CYLD mRNA, determined by quantitative RT-PCR, dropped after serum withdrawal and was recovered after re-addition of serum (Figure 4E). Already after one hour serum re-addition almost two fold CYLD mRNA could be detected (Fig 4E). To confirm this result, cells were serum deprived before re-addition of serum in the absence or presence of actinomycin D. This treatment reduced CYLD mRNA (by >50 fold) and protein (Figure 4F) both in the absence or presence of serum (Figure 4F), suggesting that CYLD gene expression is regulated rapidly at the transcriptional level by serum. In corroboration to this, the levels of cyclin D1 and its mRNA stability which is dependent on the mitogenic signaling and it is also a critical factor for actively cycling cells in the presence of serum, showed the same pattern as CYLD when serum was depleted (Figure 4A). As in the absence of serum for 48 hours CYLD but not cyclin D1 could be detected (Figure 4A), this might indicate that CYLD is a more stable protein than cyclin D1.

### The serum regulated recruitment of SRF to the CYLD promoter is dependent on p38MAPK activity

**Figure 5:**
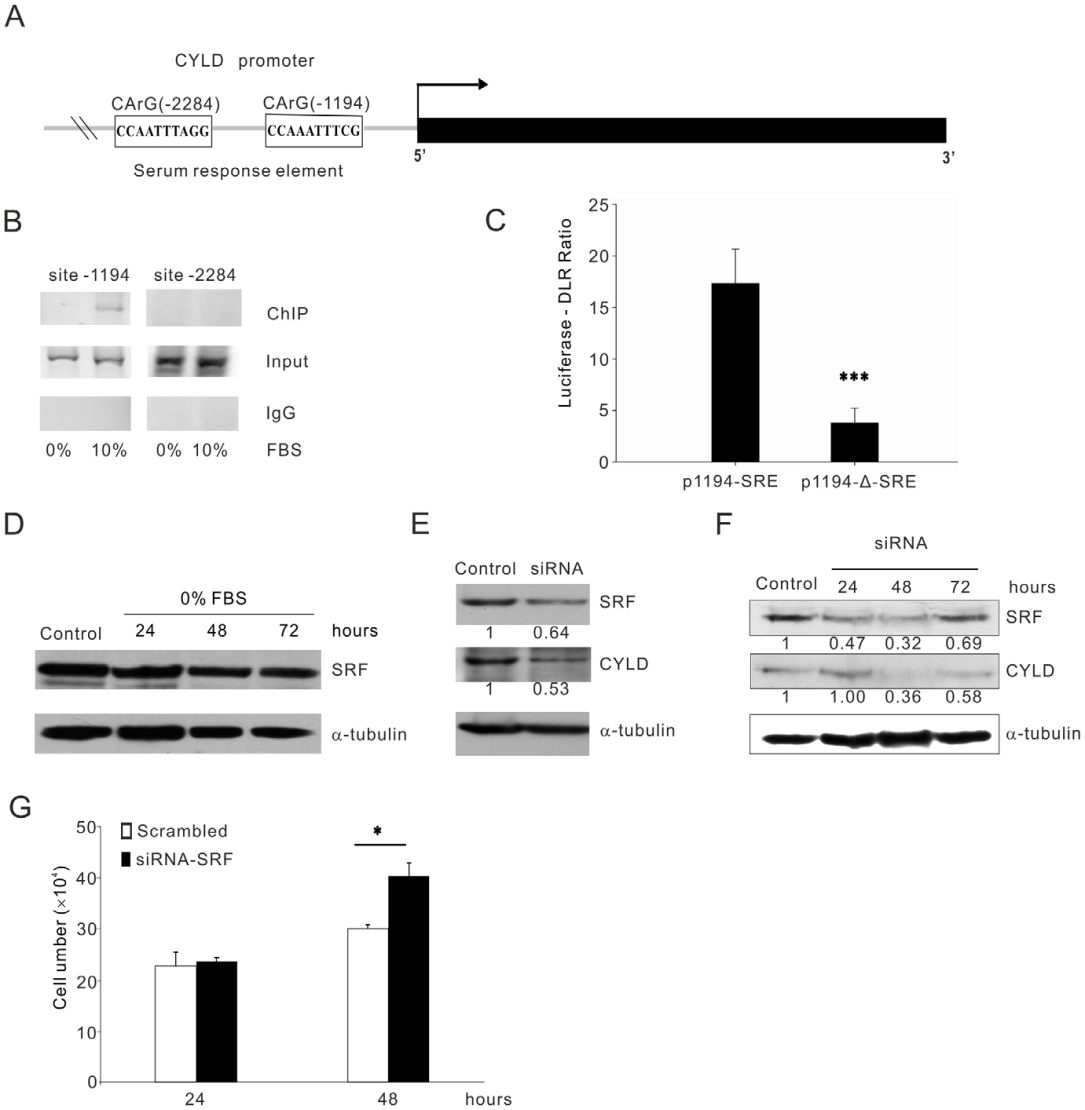
Recruitment of SRF to the promoter of CYLD induced by serum. (**A**). Location of two serum response elements identified at CYLD promoter. (**B**). Lysates from CYLD+/+ MEFs were examined by ChIP assay using an anti- SRF (H-300, Santa Cruz) and a PCR primer pair corresponding to the promoter of the CYLD gene (270 bp). Immunoprecipitation (ChIP) using specific antibodies; IgG: IP using negative control rabbit immunoglobulin; Input: 10% of the cell lysate used for the IP is shown. (**C**). Reporter assays revealing inducible CYLD promoter (-1297 to -1) activity in MEF cells; (p1194SRE), whereas mutation of the consensus SRF binding site (p1194ΔSRE) led to reduced promoter activity. (**D**). Western blot analysis of SRF and tubulin expression in the presence of 10% FCS for 48 hours (control) or cells incubated in the absence of serum over a period of 24–72 hours (0% FBS). (**E**). Western blot analysis of SRF, CYLD and tubulin expression in scrambled siRNA control transfected cells or cells transfected with the SRF siRNA nucleotides. (**F**). Western blot analysis of SRF, CYLD and tubulin expression in cells transiently transfected with the SRF siRNA nucleotides after serum withdrawal (24 hours) and re-addition of FCS over a period of 24–72 hours. Control cells are transiently transfected with the scramble siRNA nucleotides after re-addition of FCS for 48 hours. (**G**). Cell counting of CYLD+/+ MEFs transiently transfected with scramble or SRF siRNA nucleotides in the presence of 10% FCS over period of 24–48 hours.

**Figure 6:**
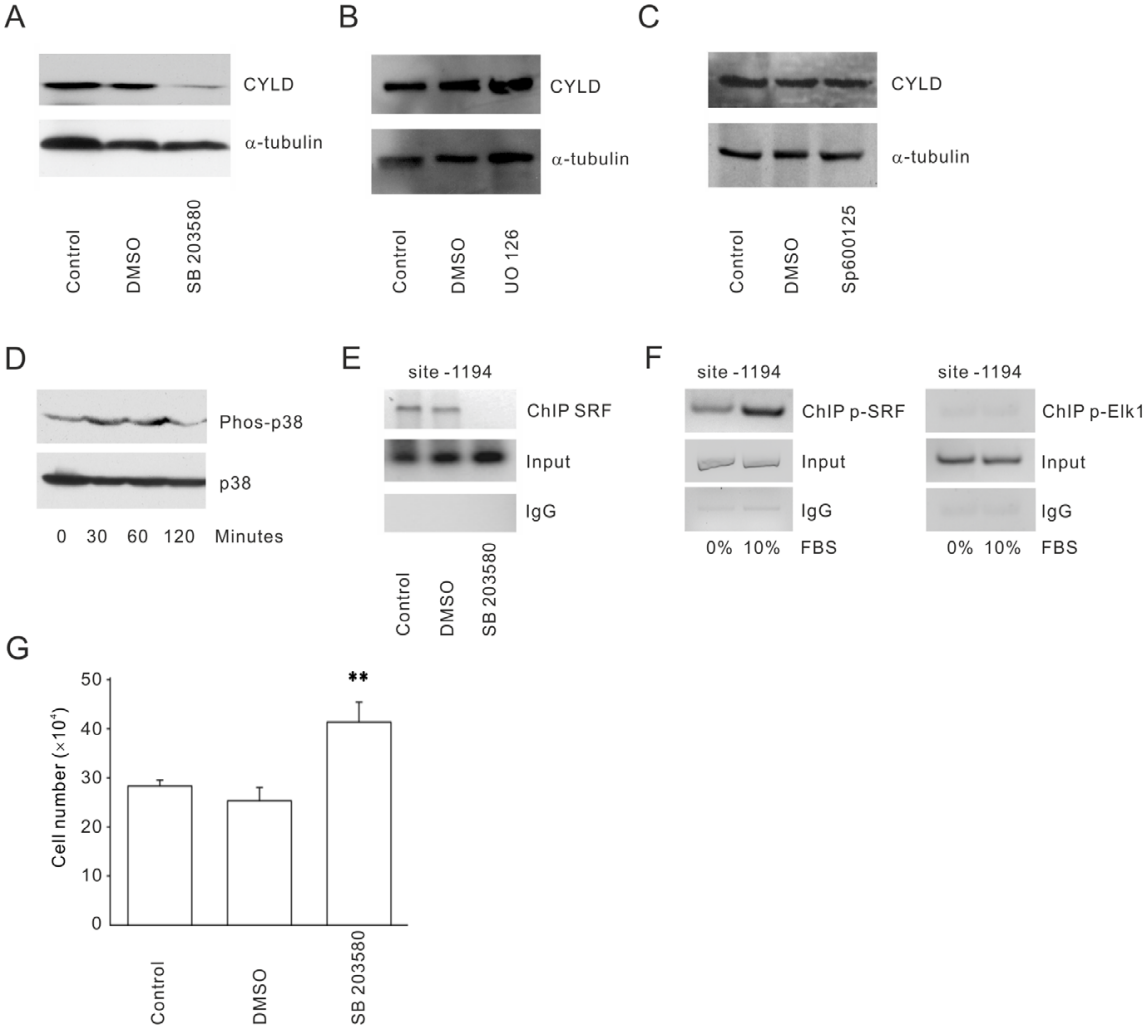
Effects of p38MAPK inhibition to serum mediated CYLD expression. (**A–C**). Western blot analysis of CYLD and tubulin expression in serum starved WT MEFs (24 hours) and readdition of 10% FCS for 24 hours in the absence or presence of SB-203580 (500 nM), PD 98058 (25 µM), UO126 (20 µM), SP600125 (20 µM) or solvent (DMSO). (**D**). Western blot analysis of active and total p38MAPK in WT MEFs in the absence (24 hours) or readdition of 10% FCS for 30, 60 or 120 minutes. (**E**). Lysates from CYLD+/+ MEFs were examined by ChIP assay in the absence or presence of solvent (DMSO) or SB203580 (500 nM for 1 hour). Cells were serum starved for 24 hours and one hour before readdition of serum, SB203580 (500 nM for 1 hour) or solvent (DMSO) was added to the cell culture. Cell lysates were chromatin immunoprecipitated using anti-SRF antibody and a PCR primer pair corresponding to the promoter of the CYLD gene was used. Immunoprecipitation (ChIP) using specific antibodies; IgG: IP using negative control rabbit immunoglobulin; Input: 10% of the cell lysate used for the IP is shown. (F). Lysates from CYLD+/+ MEFs were examined by ChIP assay using anti-pSRF or anti- pELK1 and a PCR primer pair corresponding to the promoter of the CYLD gene (270 bp). Immunoprecipitation (ChIP) using specific antibodies; IgG: IP using negative control rabbit immunoglobulin; Input: 10% of the cell lysate used for the IP is shown. (**G**). Cell counting of CYLD+/+ MEFs in serum starved WT MEFs (24 hours) before re-addition of 10% FCS for 48 hours in the absence or presence of solvent (DMSO) or SB203580 (500 nM for 1 hour).

SRF is a transcription factor that binds to the CArG box in immediate-early genes and activates their transcription after serum stimulation. Analysis of the mouse *CYLD* promoter revealed two potential CArG boxes (Figure 5A). Chromatin immunoprecipitation (ChIP) assays demonstrated that SRF was recruited to the first CArG box of the CYLD promoter at position -1194 only when the cells were cultured in the presence of serum, but not to the position -2284 (Figure 5B). Two well known factors involved in activation of SRF are serum and LPA ([29], [30]). Reporter gene assays using the CYLD promoter (-1297 to -1) revealed inducible activity in MEF cells, whereas mutation of the first CArG box of the CYLD promoter at position -1194 significantly repressed CYLD promoter activity (Figure 5C). Since serum but not LPA was able to induce proliferation of CYLD−/− MEFs (Figure 2G), we decided to investigate serum-mediated SRF activation and expression. No significant changes could be observed in the levels of SRF when wild-type and CYLD−/− MEFs cells were serum depleted (Figure 5D). This might indicate that the expression level of SRF is not altered instead activation of the SRF might be important for mediating downstream signaling. To analyze whether SRF directly influence the level of CYLD, we treated CYLD+/+ MEFs with siRNA against SRF in the presence of serum and found that reduction of SRF levels caused a significant down-regulation of CYLD (Figure 5E). Even re-addition of serum to SRF-siRNA treated cells was unable to restore the levels of CYLD after 48–72 hours (Figure 5F). To investigate whether removal of SRF, which caused reduced expression of CYLD, can mimic the proliferation phenotype observed in CYLD−/− MEF cells, CYLD+/+ cells were treated with scramble or SRF-specific siRNA. In concordance with our assumption we could show that depletion of SRF after 48 hours caused an increase in proliferation rate of CYLD+/+ cells compared to the control siRNA treated cells (Figure 5G). These results together suggest that binding of SRF to the promoter of CYLD is important for up-regulation of CYLD, which in turn reduces proliferation of MEF cells in the presence of serum. The proliferation and survival of cells in response to serum is a complex network, which integrates signals such as p38MAPK, c-Jun N-terminal kinases (JNK), and extracellular signal-regulated kinase (ERK). To investigate whether any of these signalling pathways can mediate serum-induced expression of CYLD, wild-type cells were treated with p38MAPK, ERK, and JNK specific pharmacological inhibitors. Figure 6 demonstrates that while pre-treatment of cells with p38MAPK inhibitor (SB203580) caused reduction in CYLD expression levels (Figure 6A), neither ERK (PD98058, UO126), nor JNK (SP600125) had any significant effect (Figure 6B–C). To further analyse and monitor activation of p38MAPK upon serum treatment, CYLD+/+ MEFs were serum starved for 24 hours before re-addition of serum for 30, 60 and 120 minutes. This analyse revealed an initial and transient activation of p38 at 30 and 60 minutes which declines after 120 minutes (Figure 6D). To investigate whether recruitment of SRF to the promoter of CYLD is dependent on the activation of p38MAPK, we treated cells with SB203580 and performed ChIP analyses. This assay demonstrated that inhibition of p38MAPK blocks recruitment of SRF to the promoter of CYLD in the presence of serum (Figure 6E). As p38 directly phosphorylates ELK1, and p38 downstream kinase MAPKAP (MK2) can phosphorylate SRF, we decided to find whether p38 directly or through MK2 induces CYLD gene expression. To address this question, we performed ChIP using wild type cells in the absence or presence of serum. Re-addition of serum to deprived cells, recruited phospho-SRF but not phospho-ELK to the promoter of CYLD (Fig. 6F), suggesting that p38 mediated MK2 activation and phosphorylation of SRF is vital for upregulation of CYLD. Furthermore, pre-treatment of CYLD+/+ MEFs with SB203580, which is also an inhibitor of MK2, increased proliferation rate of these cells compared to control treated cells (Figure 6G). These results together suggest that serum is an essential factor for p38MAPK mediated SRF activation, which in turn causes up-regulation of CYLD at the transcriptional level. Moreover, an increase in CYLD levels reduces the proliferation rate of MEF cells.

## Discussion

CYLD is a DUB enzyme which is lost in different types of human cancer [8]. The tumor suppressor function of CYLD in skin is due to the reduced proliferation rate mainly through a delay in the G1 to S phase transition by reducing cyclin D1 expression levels [2], [10], [31] in a NF-κB dependent signaling pathway. In addition to reduced proliferation, CYLD was shown to promote apoptosis in different cell types including HeLa [27] or HaCaT cells [28] by interfering with NF-κB signaling pathway. Beside the classical NF-κB pathway, CYLD can also interfere with JNK and p38 MAPK signaling in a stimulus and tissue specific manner [11], [32], [33], [34], [35], [36]. Although there are studies describing different signaling pathways regulated by CYLD during infection, inflammation and neoplasia [8], it is not known whether and how positive transcription regulation of CYLD on the promoter level is orchestrated in non transformed cells. In the report, we have studied cell proliferation in MEF cells, which is an extensively employed model for examining proliferation under normal conditions [37]. We have found that CYLD−/− MEFs have a higher proliferation rates than wild-type MEFs, independent of the passage number in culture, but depend on concentration of serum. However, in the absence of serum, the growth retardation is similar between CYLD+/+ and CYLD−/− MEFs. Despite the previous report showing that CYLD can promote apoptosis by interfering with TNF-α signaling pathway [27], [28], we did not observe any differences in apoptotic rate under (1) non induced condition, (2) removal of serum, (3) when the cells were treated with TNF-α alone or (4) when treated with TNF-α together with cyclohexamide. This indicates that absence or presence of CYLD in primary non-transformed fibroblast is not involved in TNF-α mediated apoptosis. The finding that concentration of serum could affect the proliferation rate of CYLD+/+ compared to CYLD−/− MEFs prompted us to investigate whether the levels of CYLD is regulated by serum. Indeed removal of serum from culture media resulted in a decrease of CYLD while re-addition of serum to the culture media transiently increased CYLD at the protein level. To determine whether gene transcription of CYLD can be regulated at the RNA level we analysed by quantitative RT-PCR, the CYLD mRNA levels upon exit and re-entry in cell cycle in response to serum at different time points. As serum withdrawal reduced the CYLD mRNA level already after one hour, serum re-addition elevated CYLD mRNA significantly. These data indicates that regulation of CYLD gene transcription is rapid in response to the serum which regulates the context of entry and exit in cell cycle progression. In line with this hypothesis, we found a significant delay in G1-to-S phase progression of CYLD+/+ compared to CYLD−/− MEFs.

Serum response factor (SRF) is a transcription factor which regulates the expression of immediate early genes and cytoskeletal components through binding to its target promoter sequence, called the CArG box. CYLD promoter analysis identified two potential SRF binding sites, and recruitment of SRF to one of these sites occurred in the presence of serum. Removal of serum however, prevented/released SRF from this CArG box. In this aspect the inducible recruitment of SRF to the CYLD promoter may exhibit an anti-proliferative while it has been shown earlier that SRF-dependent gene expression is essential for proliferation in rat embryo fibroblast, myoblast and PC12 cells [38], [39], [40]. In contrast to these studies, SRF depletion in mice is not affecting proliferation of embryonic stem cell [41] or primary keratinocytes [42]. This discrepancy could be explained by different functions of SRF depending on the tissue/cell type in combination with the requirement of a specific stimulus. To investigate whether the SRF protein amount influence the serum induced CYLD expression, we analyzed the expression pattern of SRF in the absence and presence of serum. Since no significant differences in the levels of SRF could be detected under normal serum condition or when serum was removed, we decided to deplete SRF by using siRNA. Knockdown of SRF by siRNA significantly reduced levels of CYLD, indicating that CYLD expression is regulated by SRF. Moreover, cell proliferation was significantly enhanced when the cells were transfected with siRNA against SRF, compared to the control scramble transfected CYLD+/+ cells.

Which upstream signalling is responsible for SRF-mediated expression of CYLD in the presence of serum? Analyses of different MAPK signalling molecules such as ERK and p38 or JNK upon serum stimulation in MEFs demonstrated that among these molecules, p38 activation can regulate expression levels of CYLD. To determine whether transient activation of p38 in CYLD+/+ MEFs can regulate expression of CYLD via SRF, we treated the cells with p38MAPK inhibitor prior to serum addition. This treatment completely blocked SRF recruitment to the promoter of CYLD, and was not capable of increasing the expression of CYLD. In accordance with these results, inactivation of p38MAPK before addition of serum caused an increase in proliferation rates of wild-type compared to the control treated cells. These results suggest that serum mediated expression of CYLD via SRF is dependent on the activation of p38MAPK, which can in turn reduce proliferation of MEFs.

Several mechanisms have been shown to regulate SRF activity including co-factor association, phosphorylation and regulated nuclear translocation. In recent microarray experiments, however, CYLD expression was not differentially regulated following changes in actin-MAL signalling [21], and the MAL/MRTF co-activator family is not yet known to be directly activated by p38MAPK. Additionally, we demonstrated here that, LPA which activates MAL-SRF dependent transcription failed to regulate CYLD in MEFs, suggesting that CYLD induction through p38MAPK and SRF utilizes a distinct mechanism. It has been shown that phosphorylation by the Ca^2+^-dependent CaMK [23], [43] and by p38MAPK through activation of MK2 [44] affects DNA binding of SRF. Indeed, we could detect recruitment of phospho-SRF to the SRE sites located in the promoter region of CYLD, suggesting that SRF activation by p38 and MK2 are responsible for upregulation of CYLD levels. Furthermore, anisomycin induced activation of p38MAPK can phosphorylate SRF, which further promotes expression of immediate early genes [45]. An alternative possibility would involve the activation of ternary complex factors by p38MAPK which subsequently bind DNA cooperatively with SRF [46].

The SRF signalling pathways have been linked to proliferation, cell survival/apoptosis or differentiation, depending on the tissue and upstream signaling involved. In our system SRF mediated CYLD expression acted anti-proliferative without an obvious effect on apoptosis. It has been highlighted that SRF is not involved in proliferation has been highlighted in flies where SRF controls the differentiation of epithelial-derived intervein cells during wing development [47]. Furthermore, Srf mutant mice dies at E6.5 without any proliferative defect, instead they show lack of differentiation [48]. In addition the impaired differentiation of keratinocytes or SMC due to the deletion of Srf has been previously reported [42], [49]. Conversely, SRF activation by over-expressed MRTF family members results in inhibited cell proliferation, and the SRF co activator Myocardin is a tumor suppressor whose expression is reduced in human tumors [19], [20], [21]. In summary, our study identified SRF as a positive regulator for expression of CYLD upon serum stimulation which in turn reduces proliferation rate of MEF cells.

## Materials and Methods

### Cell culture and reagents

CYLD−/− mice were generated as described previously [10]. Embryos were isolated at embryonic day 13.5 (E13.5). Genotyping was performed by PCR analysis. Fibroblast cells from individual embryos were plated and allowed to grow for 1–3 days prior to experiments. All cultures were maintained in Dulbecco's modified Eagle's medium (DMEM) (sigma) supplemented with 10% FBS, L-glutamine (100 U/ml) and penicillin/streptomycin (100 U/ml) and grown at 37°C with 5% CO_2_. All experiments were performed in parallel using equal passage number (passage 3–5) and a similar period in culture.

### Proliferation assay

Mouse embryonic fibroblasts (MEFs) were seeded in parallel into 24-well tissue culture plates at a density of 1×10^4^ cells per well in full growth medium (DMEM plus 10% FBS). Cells were incubated overnight, then quiesced in serum free medium (SFM) for 48 hours before transfer to the appropriate growth-stimulatory medium. This was either full growth medium (10% FBS) or medium containing 0%, 1%, 5% FBS. Medium was replaced every day and growth curves constructed over a 96 hours period by determining cell number in quadruplicate wells using a haemocytometer. In the growth stimulation assay, after 24 hours incubation, the growth medium was removed, and the cells were washed twice with PBS and then serum starved by continuing cell culture overnight in FBS-free medium (DMEM without FBS). Cells were either untreated (controls) or treated with 0.5% FBS medium containing EGF (10 ng/mL or 100 ng/mL), TPA (100 nM), TNF-α (10 ng/mL) or LPA (1 µM) and then incubated at 37°C for additional 24 hours.

Proliferation in the presence of different concentration of FBS was also assessed by the MTS assay in a 96-well plate with the density of 1×10^4^ cells per well and detected by the MicroELISA plate reader (FLOU-star, bmg).

### Apoptosis assay

Apoptotic cells were analyzed by detecting the gradual degradation of internucleosomal DNA with the DNA binding fluorescent dye propidium iodide (PI). In brief, cells in the culture discs were fixed with freshly prepared 4% paraformaldehyde in PBS for 4 minutes and washed three times in PBS then stained with solution (3.5 µM Tris-HCL pH 7.6, 10 mM NaCL, 5 µg/ml propidium iodide, 20 µg/ml RNase, 0.1% v/v NP-40) and incubated in the dark for 20 min at room temperature. Thereafter, cells were washed in PBS and mounted on the microscope slides by using fluorescent mounting medium (Dako) and examined under the fluorescence microscopy. A total number of 200 staining cells were counted per experiment and scored for apoptosis based on the morphology of the nuclei.

### Western blot analysis

MEF cells were placed on ice and the media was aspirated. The cells were washed once with cold phosphate-buffered saline (PBS) and harvested in cold 1×lysis buffer [50 mmol/L Tris-HCl (pH 7.4), 150 mmol/L NaCl, 1% Triton-100, 1× protease inhibitor mixture (1∶100 dilution; Sigma)]. The whole cell lysates were cleared by centrifugation (12 000×g for 10 min at 4°C) and the protein content was determined. Equal amounts of protein were loaded onto 10% polyacrylamide gels in the presence of SDS and were separated by electrophoresis. The proteins were transferred to polyvinylidene difluoride (PVDF) membranes, blocked with 5% non fat dry milk in PBS for 1 hour at room temperature and followed by overnight incubation at 4°C with primary antibodies against CYLD [10], phospho-p38 MAP kinase (Thr180/Tyr182), total p38 MAP kinase, phospho-JNK, total JNK, phospho-ERK, total ERK and cyclin D1 from Cell Signaling, serum response factor from Santa Cruz, β-actin and α-tubulin from Sigma-Aldrich,. Membranes were imaged using the chemiluminescence (ECL) reagents (GE Healthcare, USA).

### Immunoflourescence

MEF cells were cultured on glass cover slides, rinsed twice with PBS and fixed for 15 min using 4% paraformaldehyde in PBS, rinsed twice and permeabilized using 0,25% Triton-X 100 in PBS for 10 minutes. After permeabilization, the cells were washed three times in PBS and blocked in 1% Bovine Serum Albumin (BSA) in PBS for 1 hour. The primary antibody Bcl-3 (C-14) from Santa Cruz was diluted 1∶50 with 1% BSA in PBS and incubated with the cells for 1 hour. A TRITC coupled anti rabbit antibody (ab6799, Abcam) was diluted 1∶2500 with 1% BSA in PBS, staining the cells for 1 hour. Staining of the nucleus was done using DAPI (Sigma) according to manufacturer's instruction.

### Reverse Transcriptase-Polymerase Chain Reaction (RT-PCR)

MEFs were washed in ice-cold PBS and total RNA was extracted from cells followed the protocol of the RNeasy extraction kit (Qiagen). The quality of RNA was analyzed and quantified by a NanoDrop spectrophotometer (SAVEEN WERNER) and used for cDNA synthesis according to the manufacturer's instruction (QPCR cDNA synthesis kit; Stratagene). PCR runs were performed in the Mx3005P real-time thermocycler (Stratagene) performing 40 two-step cycles consisting of 95°C for 30 seconds and 60°C for 60 seconds. The following primers were used:

CYLD-forward 5′-ACAACATGGATGCCAGGTTG-3′

CYLD-reverse 5′-CCGCTAATAAAGGTCCTCTG-3′

β-actin-forward 5′-TGTTACCAACTGGGACGACA-3′

β-actin-reverse 5′-GGGGTGTTGAAGGTCTCAAA-3′

GAPDH-forward 5′- TCGTGGATCTGACGTGCCGCC -3′

GAPDH-reverse 5′- CACCACCCTGTTGCTGTAGCC -3′

The samples were analyzed and normalized by the MxPro software (Stratagene).

### Transient transfections and reporter gene assays

Transient transfection was performed by using PolyFect reagent (Invitrogen) according to the manufacturer's instructions. The total plasmid DNA used for the transfection was 3 µg mixed with 10 µl transfection reagent per 60 mm dish. Depletion of serum response factor were purchased from Santa Cruz. SiRNA duplexes (2 µM) were mixed with Lipofectamine 2000 (invitrogen), transfection reagent (50 µl) and incubated at room temperature for 15 minutes. For each transfection, 0.8 ml siRNA transfection mixture was added to 80% confluent cells in 60 mm dish. Experiments were carried out 48 hours post-transfection the cells were either counted for the proliferation assay by a haemocytometer or lysed with lysis buffer for the immunoblot analysis.

The p-1194-SRE-Luciferase vector was generated using specific primers to PCR amplify region -1297 to -1 of the CYLD gene, (-1297 KpnI Forward: 5′- AAAGGTACCGTTAAAACTGTGACACAAACTCCAC-3′, -1 BglII Reverse: 5′- TTTAGATCTTGTGACAGTAACTTTCAGAACATTG-3′). KpnI and BglII digested PCR product and vector pNFκB-Luc (Stratagene Cat. No. 631904) was ligated together. p-1194-Δ-SRE-Luciferase vector was made using site directed mutagenesis kit (QuickChange XL. Stratagene Cat. No. 200517-5) together with the following primers; 1194-Δ-SRE F: 5′-CCTATAGTCATATGTCATTATTACATAGTAGGCTATAAACCATTTTCAAATA-3′, 1194-Δ-SRE R: 5′-TATTTGAAAATGGTTTATAGCCTACTATGTAATAATGACATATGACTATAGG-3′.

All cell transfection assays were repeated three times and done in triplicates. To assay for transfection efficiency, transfections were performed with a constant amount of Renilla expression plasmid. Luciferase and Renilla activities were assayed 24 hours post transfection according to the manufacturer's (Promega) instructions.

### Chromatin Immunoprecipitation

Chromatin immunoprecipitation was performed using the QuikChIP kit (IMGENEX). Briefly, MEF Cells were cross-linked by 1% formaldehyde at 37°C for 10 min. After immunoprecipitation by using antibodies against SRF, (clone G-20; Santa Cruz); phospho-Ser103-SRF (clone #4261; Cell Signaling); or phospho-Ser383-Elk-1 (clone #9181; Cell signaling), protein-DNA crosslinks were reversed at 65°C for 4 hours. The DNA was purified with phenol/ chloroform extraction, precipitated with EtOH and re-suspended in DNase free water. Products were amplified by the Mx3005P thermocycler (Stratagene) with the condition of denaturation of 10 minutes in 95°C, followed by 35 repeats of 45 seconds at 95°C, 45 seconds at 53°C, 45 seconds at 72°C and 10 minutes at 72°C. DNA were electrophoresed through 1.5% agarose gels and visualized through ethidium bromide staining. The following primers were used:

CYLD/SRF-1194-forward 5′-TTCTGCAACATTGCCTGAAC-3′

CYLD/SRF-1194-reverse 5′-GGATCAACCAGCCACACTCT-3′

CYLD/SRF-2284-forward 5′-AAACTGTGACACAAACTCCACAA-3′

CYLD/SRF-2284-reverse 5′-CCCATCTCTTTAGGCCTCCT-3′

### Cell cycle analysis

1×10^5^ MEF+/+ or MEF−/− cells per well were seeded in 6-well plates in duplicates. The cells were synchronized for 24 hours in serum free medium before re-addition of 10% serum. After 24 hours the cells were rinsed in PBS, trypsinized, and centrifuged (400 rpm) for 5 minutes. Pellets were either re-suspended in 20 µl complete medium and analyzed using “2-step cell cycle analysis” according to manufactures recommendation by using Nucleo-Counter NC-300 (Chemometec) or re-suspended in 1 ml ice cold 70% EtOH and stored in −20 °C (1 hours). After hydration of the cells, pellets were dissolved in 0,5 ml of Vindeløv solution [500 ml DNase free water, 021 g Tris HCL (to pH 7,6) 0,025 g Propidium iodine, 0,5 ml Nonidet P40, 9,46 g Rnase, 0,29 g NaCl] and incubated for 20 minutes, and analyzed using FACSCalibur (Becton-Dickinson).

### Statistical analysis

Data represented are mean ± S.D. of at least three independent experiments. Statistical comparisons were carried out by t-test analysis and significance was indicated by P<0.05.
